# Like Taking a Magnifying Glass Into Everyday Life: Vulnerable Parents’ Experiences With Video Guidance in an Infant Mental Health Clinic

**DOI:** 10.3389/fpsyg.2021.542716

**Published:** 2021-09-13

**Authors:** Indra Simhan, Kari Vik, Marius Veseth, Aslak Hjeltnes

**Affiliations:** ^1^Department of Child and Adolescent Mental Health, Sorlandet Hospital, Kristiansand, Norway; ^2^Faculty of Psychology, University of Bergen, Bergen, Norway

**Keywords:** qualitative research, parents psychology, parent-infant, parental mental health, video guidance, video feedback, marte meo

## Abstract

**Background:** Parents are a central focus in clinical infant mental health interventions because of the key importance of the caregiver-infant relationship, especially when dyads are burdened by psychosocial and parental mental health problems. However, knowledge is scarce about the lived experience of vulnerable parents who undergo video-based guidance.

**Aim:** The study explores how parents in an infant-psychiatric outpatient clinic who struggled to mentalize and remain emotionally connected to their infant experienced helpful and challenging elements in video guidance.

**Method:** We analyzed the interviews of a strategic sample of 12 parents after undergoing Marte Meo video guidance, using a team-based, reflexive thematic analysis (TA).

**Results:** We identified four main themes: (a) Handling initial feelings of fear and loss of control; (b) Filming as a disturbing or agentic experience; (c) Feeling validated or devalued in the therapeutic relationship; and (d) Bringing insights from video guidance into everyday life. Therapeutic and existential factors became apparent in the main themes of adjustment to the guidance, experiences with filming, the therapeutic relationship and integration of new experiences.

**Conclusion:** The parents’ sense of agency, dignity, and shame may be important for their ability to integrate new ideas about themselves.

**Implications:** Video guidance for vulnerable parents in specialized clinical treatment should address relational challenges, parental mental health, and issues of recognition.

## Introduction

What is it like to receive an intervention focusing on your parenting capacity and skills when you are burdened by mental health problems and struggling to understand and connect with your own infant? Which factors help you engage in the intervention, and which are challenging? Parents are a central focus in infant mental health interventions because of the centrality of the parent-infant relationship for infant development and attachment (Word Health Organization, 2004). Parents burdened by psychosocial and mental health problems have received particular attention, as their multiple challenges put a strain on their resources and capacities as caregivers (Berg-Nielsen and Wichstrom, 2012; Parfitt et al., 2013). Focus has been most on the risk this poses for infants (Lyons-Ruth, 2008; Musser et al., 2018) and on the effect of interventions (Camoirano, 2017). There is less empirical knowledge about the lifeworld of parents who are overburdened or challenged by mental health problems and even less about their experiences of interventions that target parenting. Increased knowledge about the parents’ experiences is relevant to elucidate important processes involved in parenting under duress as well as adapt interventions to their needs. In this article, we explore how clinically referred parents who struggle to mentalize or maintain an emotional connection with their infants experience the process of undergoing Marte Meo video guidance.

Video-guidance interventions use revised film clips of the actual dyad in the guidance of parents (Balldin et al., 2018). Marte Meo, a video guidance method widely used in Europe and Australia, is empowerment-based and resource-oriented, aiming at enhancing caregivers’ intuitive parenting capacity. Only video clips showing developmentally supportive interaction are chosen for the feedback session with the caregivers (Aarts, 2008). Osterman et al. (2010) highlighted a knowledge gap between ready acceptance by counselors and clinicians in family guidance, child protective services (CPS) and infant mental health and the scarcity of scientific evidence and theoretical foundations of the method. This gap has partly been addressed by effect studies on parent-infant dyads with interactional problems (Høivik et al., 2015) and vulnerable first-time mothers (Kristensen et al., 2017). Bunder (2011) found that overburdened parents developed more positive structuring but especially more emotional connection through Marte Meo. Explorations of parental experiences of change have led to attempts to conceptualize the method in an attachment and mentalization framework (Vik and Hafting, 2009; Vik and Rohde, 2014; Gill et al., 2019).

However, since the existing studies are based in community settings, except for a pilot study including three parents from a psychiatric outpatient clinic (Vik and Hafting, 2006) and a three-case study from an infant psychiatric clinic (Gill et al., 2019), empirical knowledge about how the method works for clinical-range high-risk parents is marginal. Parents in specialized infant-psychiatric treatment are characterized by more serious or long-term mental health problems and related psychosocial stress, suffering from recurrent affective disorders, anxiety disorders, substance misuse, personality disorders, and others (Berg-Nielsen et al., 2002; Pajulo et al., 2011; Anke et al., 2019). Often, their intuitive parenting capacity is weakened (Papousek and de Chuquisengo, 2006) and they have difficulties establishing and regulating their emotional connection to the infant and discovering and mentalizing the infant’s signals (Freeman, 2016; Binion and Zalewski, 2018). Research from communal settings shows effects for parents with depression and some personality disorders (Høivik et al., 2015) and high psychosocial risk (Bunder, 2011), which is supported by qualitative studies (Vik and Braten, 2009; Gill et al., 2019). There is a need for more empirical knowledge about how parents in specialized infant mental health services experience and respond to Marte Meo guidance.

The aim of the study was to explore the lived experience of parents from clinically referred dyads with difficulties mentalizing and maintaining emotional connection with their infants. We wanted to investigate the first-person perspective of how these parents experience the use of video guidance to describe and understand potential helpful and hindering factors when using the method for these dyads. We examined the following research questions: How did the parents experience the process of participating in Marte Meo video guidance? What did they experience as helpful and unhelpful aspects of this guidance?

## Materials and Methods

This project was designed as a hermeneutical-phenomenological investigation of the lived experience of parents receiving Marte Meo guidance in specialized mental health settings. We chose team-based, explorative, reflexive thematic analysis (TA) as the method of data analysis. In TA, meaning units, patterns, and key themes are generated from the qualitative data in an analytic and interpretative process. Results are thus not “found” in the data but develop in a dialogical process between the data and the researchers. The collaborative approach helped us to reflectively use our own preconceptions when conducting the research process.

### Setting

The study was a collaboration between the Research Unit and the Infant Mental Health Team (IMHT), an outpatient service for parent-infant dyads, Department for Child and Adolescent Mental Health, Sorlandet Hospital, Norway, and the Department of Clinical Psychology at the University of Bergen, Norway. Clinicians from the IMHT assisted in the sampling process and administered Marte Meo guidance to the participants.

### Data Collection Strategy

We used criterion sampling (Sandelowski, 2000) to recruit a strategic sample of parents of infants referred to the IMHT who had difficulties mentalizing or maintaining an emotional connection with their infant. Recruitment was assisted by the Parent Development Interview, revised version (PDI-R; Slade et al., 2004, Unpublished),^1^ with the criterion being either (a) limited reflective functioning (RF) in the PDI-R, or (b) explicitly stated difficulties emotionally connecting with the infant and maintaining the connection under affective stress. The strategically recruited parents each received a course of Marte Meo video guidance. After the guidance process, they were interviewed individually about their experiences by in-depth interview. As we understood the phenomenological-hermeneutic position of reflexive TA as inconsistent with the concept of data saturation (Braun and Clarke, 2019b), we employed the concept of information power (Malterud et al., 2016) and aimed at a sample size of 10–15 participants.

### Parent Development Interview

Clinicians from the IMHT administered the PDI-R to 30 consenting parents. The PDI-R is a 45-item semi-structured narrative interview about parents’ representations of their children, themselves as parents, and the parent-child relationship. A subset of 12 items can be rated for RF, defined as the manifestation of mentalization in speech or the capacity to recognize and understand internal experience in terms of underlying mental states (Slade, 2005). Interviews were digitally recorded and transcribed verbatim. The first author, a certified rater, read them for content and rated the subset of items on the 11-point RF-scale from limited to moderate to high RF (Fonagy et al., 1998); an overall score of less than five represented limited RF. Thirty percent of the PDI-R were double-coded by two certified, blinded external raters. Interrater reliability was good with an intra class correlation coefficient range from 0.75 to 1.0 (Koo and Li, 2016). Parents with an RF of four or less and parents with an RF above four who explicitly stated profound difficulties maintaining an emotional connection when interacting with their infant were recruited as participants.

### Participants

A strategic sample of 15 parents was recruited. Two parents withdrew consent after the guidance, and one was not included because the infant was removed from care during the guidance process. We conducted in-depth interviews with 12 parents, 11 mothers and one father between 23 and 34years of age (*M*=27). All recruited dyads were referred based on the risk to the infant linked to parental functioning or mental health problems. Infant sex was equally divided between male and female, and infant age was between 2 and 30months (*M*=12months) at the start of the guidance. All parents reported mental health problems, mostly of a long-term or recurrent nature, including personality disorders, post-traumatic stress disorder, uni- and bipolar affective episodes, obsessive-compulsive disorder, and substance misuse disorder. Most of them reported adverse or traumatic childhood experiences. In four families, the CPS were involved. Ten parents showed limited RF in the PDI-R, while two reported substantial difficulties in experiencing a stable emotional connection with their infant.

### Video Guidance

Marte Meo video guidance (Aarts, 2008) was tailored to each individual dyad, with at least three filming and guidance sessions. Marte Meo therapists receive 2-year part-time, supervised training, and certification in the method. Four Marte Meo therapists from the IMHT with extensive experience with parent-infant dyads administered the guidance.

### In-Depth Interviews

We devised a semi-structured interview guide to assist the in-depth interviews. Example questions were: “Is there something from the guidance you specifically remember?”; “How did you experience filming?”; “How did you experience the therapist? Was something he/she did helpful or not helpful?”; “Concerning your thoughts about the infant, has the guidance changed them in any way?” We used the guide to structure the interviews but encouraged participants to pursue topics they found relevant. All interviews (*n*=12) were conducted by the first author between November 2016 and June 2019 and lasted between 23 and 89min (*M*=61min). The interviews were digitally recorded and transcribed verbatim.

### Methodology

We chose explorative, reflexive TA as a method allowing an inductive, data-driven analysis (Braun and Clarke, 2006, 2019a). We aimed at a reflexive, experience-near reporting of the data and carried out the analysis as a team-based approach, which further strengthened the balance between closeness to the participants’ experience and reflecting on our own position as researchers (Binder et al., 2012).

### Data Analysis

Reflexive TA is a stepwise process of data analysis and interpretation, moving from single meaning units, or codes, *via* shared meaning patterns to key categories, or themes. We used NVivo 11 qualitative data analysis software (QSR-International, 2015) for technical assistance in organizing meaning units and patterns. Reflexive TA was carried out in a collaborative process by the first, third, and last author: (1) All collaborators familiarized themselves with the data and noted their first impressions and reflections about the experience related to each interview. (2) The first author reread each interview, identifying meaning units and generating 52 initial codes. Meaning units were understood as features of the data that appeared interesting or seemed to convey meaning regarding the phenomenon. Existing codes were used across interviews only if they were considered a suitable description. (3) The first author reported the coded meaning units back to the group, and 19 meaning patterns and four main themes for the meaning units were formulated in a collaborative process. (4) The first author and the group summarized and reviewed themes in a back-and-forth process to define the most important and relevant themes. (5) The first author refined the themes and wrote an analysis of each theme. (6) The themes were drawn together in writing, related to the research questions. (7) The research team formed a consensus on the formulation of the four main thematic categories.

### Researchers

The first author, a child psychiatrist, and the second author, a sociologist, are Marte Meo video guidance therapists specializing in infant mental health. The third and last authors are associate professors in clinical psychology. All authors have extensive clinical experience with psychotherapy and other mental healthcare approaches, as well as experience with qualitative research on a range of topics in mental health.

### Ethics

The study was approved by the Regional Council for Research Ethics, Southeast Norway. Clinicians at the IMHT gave oral and written information about the study to suitable parents and obtained written informed consent for participation and publication from all participants. The researchers were aware of the vulnerable position of both parents and infants involved in the research and were actively concerned with preserving the dignity of the participants in the interviews and subsequent research process.

## Results

We organized the parents’ experiences of helpful and challenging factors in the video guidance process into four main themes: (a) Handling initial feelings of fear and loss of control; (b) Filming as a disturbing or agentic experience; (c) Feeling validated or devalued in the therapeutic relationship; and (d) Bringing insights from video guidance into everyday life.

### Handling Initial Feelings of Fear and Loss of Control

The first theme illustrates the participants’ emotional experiences at the start of the guidance. Most parents felt burdened by mental health issues and found it hard to cope as caregivers. They describe agency and choice, as well as their own psychological problems as factors that influenced how they initially felt. How free or coerced they felt and how much trust they placed in the intervention, made them feel less or more agentic. Many expressed the wish for more practical information, which would have helped them prepare for the guidance and counteracted feeling a loss of control when meeting the therapist who had all the knowledge. Some parents described themselves as agentic from the outset, albeit somewhat apprehensive. For most, however, this was a more conflictive issue. Disagreement with the need for referral was handled in different ways; it could create distance but also prove to be a positive challenge:

“When [the clinician] read [the referral] out loud, I felt like, no, it is not like that, I am not like that. [Begins to cry] I felt really unwell, I thought ‘Am I so awful?’ … I was unaware of what was written in the referral. I came in, in a good mood and positive, and then … ‘No way’. I was not going to accept that, and I felt like, I will agree to this because I do not want to be seen like this, I do not want them to think that I am like this. I want to show them they are wrong. That I can do much better than that.”

Involvement of the CPS played a role but was not decisive; they could be seen as both controlling and serving the parents’ needs. Parents who were motivated from the outset could distinguish between CPS involvement and the video guidance: “We felt [the Infant Mental Health Team] were there more to maybe help us, not jail us …. Help from [them] was wholly voluntary, because we ourselves had asked for it.” However, it could be a painful process for parents to experience concern for their own infant and realize that they needed help:

“We felt we were not good enough as parents right now, we need more help. … We really want what is best for [the infant]. It is tough – we reported ourselves to the CPS, that was heavy… we could have refrained from it, but then it would merely have been a matter of time before everything went wrong … because we know we are struggling.”

In the midst of crisis, this parent nevertheless conveyed a sense of agency and an intrinsic confidence that help could be found. Several parents described an inclination to trust and gain new insights, and some even expressed curiosity about the guidance. For others, however, the intervention felt threatening or even overwhelming, involving a loss of control. They described a high degree of apprehension, a greater need to feel secure, and intense feelings of inadequacy. One parent felt constrained and paralyzed by her own inner pressure and fears, which she described as part of her psychological burden. She agreed to the guidance – both despite and because of her fears:

“My whole approach to … being sick has been about my inability to make decisions. I do what I am told to. So, when my therapist at [psychiatric clinic] suggested this could be a good idea, I say yes. Because the alternative would be to say no, and then maybe they write in my medical journal that I refused, and confront me with it later, that I was offered this treatment, but you just say no. So, this is not about trust, this is about an enormous fear that they could use that against me later …”

She masked her fear and did not think her video guidance therapist realized how afraid she truly was; “I have a very well-functioning autopilot … Despite a very poor level of functioning [inside], I can function very well on the outside.” She described an inner struggle between coming to terms with her psychological problems and feeling fear and despair of being defined by them. Several parents described that accepting help was a process requiring time. One parent relayed how she understood that she needed support to obtain better mental health and cope with parenthood. However, while she felt increasingly worn out, she could still not force the decision:

“I realized that I could not sort this out by myself … it was important to get help, and for that, I needed to open up. The problem was, I should have tried to get help much sooner … I was so exhausted and so tired … I should have opened myself more, I know that – but at the same time, I needed to build up enough security to do it …”

### Filming as a Disturbing and Empowering Experience

The second theme describes the parents’ experience of being filmed and seeing themselves on film during the guidance. All parents described film, the central medium in video guidance, as challenging, even though they also found it empowering. While they noted that much of the focus was directed toward the infant, they became acutely aware of themselves during both filming and reviewing. They described feeling self-conscious, inauthentic, and apprehensive about losing control in the review situation. Often, they felt unnatural when filmed interacting with their infants. They tried to perform well, while wondering whether the video would capture something genuine. Filming created a gap, with the parents simultaneously engaged in the interaction, while at the same time thinking about being filmed. They also described an initial distance toward seeing themselves on film. Many parents said they at first did not recognize themselves in the video: “That is not me, that is a completely different person.” One parent described how she truly felt she was looking at herself only at the very end of the guidance process. The reliable focus on successful interaction enhanced this distance. All parents noted this positive stance, and nearly all found it encouraging. However, many struggled with acknowledging that what they saw were genuine, valid interactions, that it was “real.” Acceptance was more difficult when parents had strong feelings of self-doubt and criticism:

“I cannot believe in the positive. I cannot believe that this is me, or I believe that this is just accidental, or that it was just because it was on film, or that I was just because I was trying to do my best. That I am not really as good normally, that it is more just performance. So, I only see the negative.”

Many parents noted the attention that filming gave to minute interactions between the infants and themselves; “these little details … that you normally would not observe or particularly think about.” This felt like a novel way of seeing. When small exchanges were magnified and highlighted, this focus also conferred weight to the parents. This could feel uplifting and empowering but entailed challenges: “You see that what you do … is important … [but] even if it is positive, you feel a lot of focus on all the things you do.” Most felt themselves uncomfortably scrutinized; some felt as if they were under surveillance and experienced this as threatening. For parents who struggled with feeling very insecure and self-critical, being minutely observed seemed to impinge more upon their personal space and could feel embarrassing and intrusive:

“Scary, actually … because there is so much focus on you and the child…. Because you are made very aware of everything you do. All the ways in which you move, the glances you direct, your body language … All those small things are noted a great deal. … It is almost more about myself than about the child. It feels scary because it is new and strange. It feels out of my control, anyway.”

Nevertheless, almost all parents related positive development; many even felt empowered. In the course of the process, most of them were able to let go of the discomfort and worries about their own shortcomings, connect with what they saw on film, and increase their awareness of the interaction. One participant described this inner journey:

“It was very strange. In a way, I felt this was not me. Well, I saw it was me, but … I experienced the interaction between me and [the infant] on film as very different from what I had thought it was. … I did not think it was genuine, you know, outside of the film. [But] I started feeling more confident about myself, that maybe I really am like that. [On film] I looked at him a lot, and smiled, and was in contact with him … And after the guidance, I have been very aware about being in contact. Eye-to-eye contact and that he looks at my face … And I feel I was being myself somehow. I did not feel I was merely acting either, so I was actually being myself.”

Another parent who had struggled with profound feelings of insecurity and insufficiency as a mother described the transition to feeling more agentic and being able to handle her own self-doubt:

“It was really awkward. To see myself on film … I thought, okay, this is going to be really terrible. I was sure I had done everything wrong … But in the middle of the film, when I saw so many positive things, I thought, I am just a mom, after all. I am just an ordinary mom who tries her best, so there is nothing to be afraid of. The film is just there to help me, nothing else. It is not going to be shown at the cinema, after all.”

### Feeling Validated or Devalued in the Therapeutic Relationship

The third theme describes facilitative and conflictive experiences with the therapist and the therapeutic relationship. Most parents expressed that the therapist was important in “giv[ing] the parents confidence that … we are here to help you, not put any more spokes in the wheels.” It was also important that the therapist took time to establish contact with the infant and show interest in the parent as a person. Even though, the guidance was primarily focused on their relationship with the infant, most parents also wanted it to be about themselves as persons, about their feelings and experiences:

“…That it was about myself as well. Because, no offense, but with the health visitor, everything was about the baby. The baby’s physical health, and so on and so on. Yet it is just as important that the mother is doing well, mentally anyway. So, for once, I felt that *I* was being taken care of.”

Most parents described this wish to be acknowledged and to be seen as a human being in their own right. Moreover, it was important for them to feel accepted as competent caregivers who needed help with a defined problem and not to be seen as entirely defective. Parents also felt validated when the therapist respected their boundaries:

“[It was helpful] that she actually talked to me and understood [our] past history. That she had time for that. That … she even understood if she was not welcome …That she did not just do her job. That she was more a person, not just somebody who did their job.”

The therapist becoming visible as a human being was the other side of this picture, though the parents differed in the way they thought about this and how important this was for them. For most, experiencing common humanity with the therapist strengthened the therapeutic relationship. However, parents who struggled with their self-worth could compare themselves unfavorably and experience feelings of insecurity and inadequacy. It was reassuring but also daunting when the therapists showed unfailing competency. Therefore, it could feel good when they not only shared their own parenting experiences but even revealed their own mistakes: “You feel validated that it is normal to struggle the way you do and that even the person supposed to be a therapist also has problems and is not a super-human being,” as one parent put it. Another parent, who struggled with feeling objectified and belittled by the guidance, described how the positive stance of the therapist felt alienating and frustrating to her:

“She is so extremely positive … everything is, like, I feel she is looking at everything through rose-tinted glasses. She is either being very professional or just a very happy person, at peace with herself. Which I am not. […]”

The same parent felt an almost persistent emotional distance toward the films, while soon catching on to what she perceived as the behavioral content of the guidance; “when I figured out how it worked, it was like … two sessions would have been sufficient. The third one was merely, ‘do this and this and that’, and I’ve got it.” When the therapist showed her videos of close interaction with the infant, her emotional distance did not diminish, while she felt applauded for something that was not worth mentioning. To her, this felt ridiculous and devaluating:

“I feel that she talks to me like I was, well, small, or ignorant. Ignorant maybe. A lot of ‘how do you feel about this?’ and ‘what do you think when you see that?’ kind of stuff. So, I just like, that is so obvious. … and then, when I answer that which I think is totally obvious, but which I realize is what she wants me to say … she goes like, “Oh, wow”. Like, if she is impressed by that she must think I am mentally retarded.”

However, most other parents described the reflective stance as very helpful. When their attention and curiosity was guided toward the infants, it helped them to let go of their own thoughts and doubts about themselves and stimulated reflection over their infant and the interaction:

“I saw so clearly how I was affected by her. I was very stressed and then I tried things that were obviously not right for her just then …But [the therapist] asks the kind of questions that make me think about what I did and the choices I made … ‘is there something else you could have done [in this situation]?’, and questions like that. And that makes me reflect more about it … [By myself], I only would have seen what I did wrong. But in [the therapist’s] presence I get to hear all that I did that was very good.”

The therapists’ attuned support was also important in the reviewing situation: “When [the therapist] saw I was stressed, she said ‘just do not think about it, just look at [the infant]’, ‘look at the way he looks at you.’ Like that. That made me relax more”. Several parents emphasized how the repeated experiences with the therapists’ reliably positive and calm presence were instrumental to develop more confidence:

“You become a bit quieter because you are aware that [the therapist] is present all the time … she repeats it … [the therapist] has a positive focus all the time, and she repeats that until you feel secure about it. You get to feel very secure about [the therapist] as a person also, because she is very calm and … does everything she can to make you trust that she will not do anything negative.”

### Bringing Insights From the Video Guidance Into Everyday Life

The fourth theme describes the parents’ reflections on the guidance process and their experiences with putting it to use in their everyday lives. A central topic was the positive stance with its predominant focus on supportive interaction. Most parents had become distinctly aware of this because it collided with their negative expectations and felt new and even upsetting. They not only had negative ideas about themselves as parents that made it difficult to accept a positive stance but also felt that the problems in the interaction should have received more attention. Should the guidance not also inform them about what they did wrong? Could a positive review be trustworthy? Was it legitimate to let go of their own inner criticism? However, they remembered previous experiences with external criticism and how this had resonated with their own self-criticism. A focus on more problematic exchanges with the infants would have supported previous convictions of their own shortcomings and pulled them down:

“[It is important] that the reviews are a positive experience … Because that makes me go out of the guidance and feel that I am doing a good job. That I can manage and that I can do a still better job. And that gave me enthusiasm, which again made me more open to see and understand…And to understand is so important…it makes you want to do it. That you don’t go out of there thinking ‘they just think all I did was bad’.”

Many parents described how they grappled with the experience of an external, reliably positive stance, represented by the therapist and the films, as opposed to their internal critical and self-doubting stance. The process of accepting and integrating could be intense and extend beyond the guidance session, requiring support from others: “I often need a lot of time afterward to digest everything … to accept it as positive … that can be difficult to handle if you do not have someone to talk to … To get confirmation from several people.” The new perspective gave assurance through one’s own visual and emotional experience, not through being told by somebody else. The pictures from the films were described as concrete proof. However, when the parents managed to connect with the positive perspective on the interaction, and themselves as parents, this also seemed to linger with them as a relational experience: “Even though you are at home in your own bathroom or bedroom or living room, there is this third person. So, you get this feeling of a presence [of another person].” Repeated experiences with the calming and benevolent presence of the therapist gave parents more confidence and reflectiveness:

“I feel more secure in my role [as parent]. Well, not all the time, but I have become better at calming myself down and thinking more reasonable again, that I am good enough, and that I am doing what I can for [the infant], that I am aware of [the infant].”

Several parents also related that they could transfer these experiences into other relationships, with their other children but also with their partners, making it easier to deal with difficult emotions or conflicts. Many described an increased capacity to look at the small things in the interaction and experience change in the mundane tasks of everyday life. The guidance had enhanced their significance as parents and they, in turn, saw more significance in small exchanges. “It is like taking a magnifying glass into everyday life and to use it, to see many more details.” Even a parent who said that she had not truly learned anything new that the guidance had strengthened her sense of the meaning of the minutiae of the interaction: “You are [usually] not aware of that because it is such an everyday thing. We do it a 100 times a day. But it is special.” The parents described a connection between their awareness of the little things, their capacity to reflect more about the infants’ signals, and their enhanced capacity for self-compassion:

“[By] how she looks at me, and her facial expressions, I realize that she communicates with me just like I with her. So, I must stop being self-critical … I must stop and try to understand [her] better … instead of … berating myself that I cannot give her what she wants. And that can be a little thing; maybe she just needed to lie close to me.”

Another parent also related the discovery that ordinary, everyday interactions could be highly important in contact with the infant:

“It makes me more aware and more able to understand what she actually needs … And that it does not have to be so much fun and action. It can be very quiet-like, just sitting on the floor together. That this can actually be enough.”

## Discussion

We identified four main categories in high-risk parents’ experiences of challenging and helpful aspects of Marte Meo video guidance.

The first theme, “handling initial feelings of fear and loss of control,” shows how many participants entered the guidance with high levels of anticipation and inner conflict. Parental fears have been scarcely addressed in the existing literature on Marte Meo guidance and not for clinical samples. In community samples, they are described as normal stress before the intervention (Clarke et al., 2011) or as a manifestation of power differentials (Kiamanesh et al., 2018). In our clinical sample from infant mental health, apprehension seems more connected with parents’ struggles to come to terms with how their psychological burden affected them as caregivers. They mainly described this as a solitary process unrelated to the therapist or even the referring agency. This perspective has not previously been described in the literature on Marte Meo guidance.

The second theme, “filming as a disturbing and empowering experience,” illuminates the parents’ experiences with video as a medium in therapy. Filming and viewing activated fears of being scrutinized and found wanting, especially in insecure and self-critical parents who may have been more prone to feel shame in the guidance situation. However, the process also created a gap between what parents thought about themselves and what they saw on the screen, gradually allowing them to integrate new viewpoints of themselves. Earlier research stressed the “outside perspective” of Marte Meo as facilitating the reflection and integration of new schemas of being with (Vik and Hafting, 2009; Vik and Rohde, 2014) or as a “surprise to the unconscious” (Steele et al., 2015) mediating change in the face of powerful emotions including shame. Our findings may be understood as first-person descriptions of these processes.

The third theme, “feeling validated or devalued in the therapeutic relationship,” illustrates how parents experienced issues of being valued and recognized for having personal worth as important in the therapeutic process. In this, they described the therapeutic stance, and being acknowledged as sharing a common humanity with the therapist, as especially important. While recognition in the therapeutic relationship has been emphasized (Vik and Hafting, 2009), issues of value and dignity have not been reported to be an important factor in previous Marte Meo research. They transcend mere method-related factors toward a broader therapeutic perspective (Gelso, 2002) and the need for recognition as an existential condition. Recognition can be seen as part of the parents’ struggle to remain a person in a potentially alienating and reifying process (Falkum et al., 2011). It may also be viewed as related to issues of shame, as a need to balance the narcissistic wound of being found wanting as a parent. This connects to findings from the second theme and could be related to studies of narcissistic personality traits in parents hindering the effect of video guidance (Høivik et al., 2015).

The final theme, “bringing insights from the video guidance into everyday life,” shows how repeated exposure to positive film clips in conjunction with the repeated relational experiences with the therapists decreased the impact of self-conscious emotions and increased the parents’ capacity to regulate themselves, reflect more, and feel more self-compassion in their daily lives. Our findings suggest this process to be a close interplay of the filming and viewing and of experiencing the regulating presence of the therapist. This expands earlier theorizing that had stressed the viewing (Vik and Hafting, 2009) and the therapeutic relationship (Vik and Rohde, 2014; Hawellek, 2015) but had not conceptualized the interaction between these elements.

Our overall findings indicate several important clinical and ethical challenges of Marte Meo guidance for high-risk parents in specialized clinical treatment. Centrally, the parents’ psychological and psychosocial burden permeates their experience of the guidance. While more psychosocially resourceful parents in earlier studies looked upon Marte Meo as a type of training or learning program (Osterman et al., 2010; Clarke et al., 2011), the vulnerable parents in our sample described the process as centrally also about themselves. Coming to terms with the existential position of struggling with mental health and as caregivers and needing guidance was an important yet mostly lonely initial process for them. The need to be recognized and validated can be seen as related to this experience, while it can also be understood as the navigation of self-conscious emotions including shame (Zaslav, 1998). The experience of especially one parent, who, while emotionally disconnected, felt devalued by the reflective stance, may point to the possibility that Marte Meo may be less useful to parents handling shame more by devaluing others than devaluing themselves. Earlier research on the usefulness of the method for depressed parents and parents with dependent and paranoid but not narcissistic personality traits (Høivik et al., 2015) would support this hypothesis. Our findings also provide a novel first-person description of the integration of new schemas of being with (Stern, 1994, 2004). The integration takes place in the conjunction of inner distancing created by video use and the experience of film as concrete proof. This description may denote that video opens up an inner space, where concrete positive pictures of relatedness can replace earlier, concrete negative pictures of possibly pre-mentalistic origin (Freeman, 2016). Crucially, this process is described as embedded in the regulating presence of the therapist, which lingers after the guidance.

### Reflexivity

Reflexivity involves how the researchers’ own background, preconceptions, and subjectivity might inform and influence the acquisition, analysis, organization, weighing, and interpretation of the data (Alvesson and Sköldberg, 2009; Binder et al., 2012; Braun and Clarke, 2019a). Aiming at an experience-near analysis of the parents’ experiences demanded reflexivity on the side of the research team. The first author is an infant psychiatrist with a background in video interaction guidance, and her interest in the parents’ lifeworld developed out of clinical work. This had both the potential to support the acquisition of relevant data, but also to color participants’ experiences by imposing preconceptions derived from her own experiences with parent-infant dyads. The analysis process was therefore critically moderated by the last author and audited by the third author, who had experience with therapeutic processes but not infant mental health nor video interaction guidance. Conceptualizing, including conceptualized language, was consciously set aside until the discussion of the findings. Since the second author was one of four Marte Meo therapists giving the interventions, she took part in the development of the study design and the final discussion of the results but not in the data analysis.

### Limitations

There are several methodological limitations in the present study. Our participants, while all recruited from a specialist clinic and striving to mentalize and connect emotionally, were heterogeneous regarding their mental health problems and psychosocial resources. Transferring our results to other parent or patient populations should be viewed with care. We used a narrative interview, the PDI-R, to strategically recruit the parents, and it has methodological limitations. It can, for example, be argued to mainly address mentalization in speech, neglecting its embodied manifestations. Our qualitative approach investigated the parents’ subjective experience of Marte Meo video guidance. It cannot answer questions regarding causal relationships between the described phenomena. We interviewed a strategic sample of 12 participants, which again raises the question of the generalizability of the findings to other contexts and populations. More idiographic approaches may have been better suited to capture the complex lived experiences of the individual parents during video guidance. Among our participants, women were overrepresented, with the risk of a skewed description of parenting experiences.

### Implications for Research and Clinical Practice

The findings in this study have implications for research and clinical practice. First, more knowledge is needed about Marte Meo for clinical high-risk samples, which should also inform and change clinical practice with these dyads. Second, research on clinical samples should cover studies on the effect of the method as well as on examining parents with different mental disorders, including personality traits related to self-conscious emotions (avoidant, narcissistic) and personality disorders. Additionally, more research on fathers as a parent group would be important. Third, future research should also investigate embodied aspects of mentalization. Moreover, future research should also cover dyads with older children of high-risk parents with more deeply engrained patterns of interacting and more developed verbal communication.

For clinical work, our findings indicate that it may be important to recognize these parents as a distinct group who experience specific and complex challenges and individual needs. For them, video guidance is closely interwoven with their psychosocial health and vulnerabilities, a psychotherapy of the parent-infant relationship more than a training program. This means that therapists need to be aware and able to handle this, with consequences for practice and training. Furthermore, this implies a closer initial assessment of the parents, and addressing issues of struggling, acknowledgement, and self-conscious emotions during guidance. Cooperation with the parents’ own psychotherapist should be considered. Finally, a further development of the method could incorporate more explicitly mentalization-based techniques.

## Conclusion

The aim of the study was a hermeneutical-phenomenological exploration of the lived experience of parents who struggle to mentalize and remain emotionally connected with their infant with helpful and challenging elements in video guidance in an infant psychiatric setting. We identified four main themes in the parents’ experiences: (a) handling initial feelings of fear and loss of control; (b) filming as a disturbing and empowering experience; (c) feeling validated or devalued in the therapeutic relationship; and (d) bringing insights from video guidance into everyday life. Our findings show that parents’ experiences of agency, dignity, and shame are important for their ability to benefit from guidance. The integration of new ideas about themselves as parents is achieved in close interplay of video and relational experiences. Therefore, video guidance for parents in specialized clinical treatment needs to address relational challenges, parental mental health, and issues of recognition in the therapeutic process.

## Data Availability Statement

The datasets generated for this study are available (in Norwegian) on reasonable request to the corresponding author.

## Ethics Statement

The studies involving human participants were reviewed and approved by the Regional Council for Research Ethics, Southeast Norway, registration number 50546. The patients/participants provided their written informed consent to participate in this study.

## Author Contributions

IS, KV, and AH contributed to the conception and design of the study. IS carried out the data collection and wrote the first draft. IS, MV, and AH contributed to the data analysis. KV contributed to the discussion. All authors contributed to the article and approved the submitted version.

## Funding

The study received set-up funding from the Norwegian Research Council through the Regional Research Foundation Agder, grant no. 222931. The main funding of the study was received by Sorlandet Hospital HF, Research Department.

## Conflict of Interest

The authors declare that the research was conducted in the absence of any commercial or financial relationships that could be construed as a potential conflict of interest.

## Publisher’s Note

All claims expressed in this article are solely those of the authors and do not necessarily represent those of their affiliated organizations, or those of the publisher, the editors and the reviewers. Any product that may be evaluated in this article, or claim that may be made by its manufacturer, is not guaranteed or endorsed by the publisher.

## Footnotes

^1^Slade, A., Aber, J.L., Bresgi, I., Berger, B., and Kaplan, M. (2004). “Parent Development Interview, Short Revised Version (PDI-R). Unpublished protocol.” The City University of New York, NY.
